# Should Civilization Suppress Quackery?

**Published:** 1894-02-10

**Authors:** 


### The Hospital

An Institutional Journal of

The Medical Sciences and Hospital Administration, with

AN EXTRA NURSING SUPPLEMENT.

Vol. XV.—No. 385.] [Feb. 10, 1894.

### Should Civilization Suppress Quackery?

Unlicensed medical practice either makes for the public good or it does not. By this standard it is to be tried; and by this standard it will in the long run either stand or fall. As medical men we have no taste for "kicking against the goads"; and as educators of public opinion we accept the challenge thrown down to us by a portion of the public, and we defend this general position—that only men of special education and training are competent to deal properly with bodily diseases. If this position can be successfully maintained, it follows in the strictest logic that only men of special education and training should be allowed by law to practise the medical art or any part of it. The mere penalising of the unlicensed use of medical titles is a delusion and a snare.

One might naturally suppose *a priori* that no intelligent person would think that a practitioner without special training could be as competent as a practitioner with special training. But in actual experience we find that a considerable percentage of the population put their trust in persons who have had no medical training. Now we cannot at any length argue this point here, for want of space, although we think it is well worth arguing. But let us put a single case, which will illustrate the whole position. There are in Central Africa medicine men, rain makers, witch doctors, and the like. Would any sane English man or woman think of putting himself under the care of such persons in a dangerous illness if an English doctor were at hand? Most assuredly not! But why not? The sole reason is that the African has no certain knowledge, and therefore no assured skill. The white doctor has knowledge, and therefore skill. It is the possession of proved knowledge and skill, and that alone, which makes the white doctor trustworthy as a doctor. It is the want of such proved knowledge and skill which makes the savage dangerous. But in this country a person who has had no medical education is necessarily as ignorant of medicine as the African witch doctor, and in many cases even more ignorant. An untrained man who practices medicine is likely to know much less of his art than an untrained man who practises law; and for this reason—the things connected with a knowledge of the law are to some extent a part of everybody's general education. But anatomy, pathology, chemistry, the medical art—none of these constitute any part of general education. So that, for practical purposes, any man who has had no special training for medicine is just as ignorant of the art and science which he essays to practise as if he had no education of any kind; just as ignorant, in short, as the Central African medicine man. This illustration may stand as a sample of the line of argument which should be addressed, not indeed to the intelligent public, but to that portion of the well-meaning public who, with the most peculiar mental perversity, insist that for life-saving purposes a lifeboat which has been specially built to float in storms is not so trustworthy as a washing-tub would be, which is not a boat at all.

The Harness case is, of course, the occasion which has led us to deal with this subject at the present time. Why should Mr. Harness not be allowed to practise medicine if he does good? And, according to some of his witnesses, he has done them good. For reply we may answer, Why should we not put half-a-dozen planks inside the boathouse instead of a lifeboat? Planks have been known to do good! We might get together quite a number of witnesses who would testify that their lives had been saved by planks, because the planks had the power of floating. But is there even a stupid sailor anywhere to be found who would prefer a plank to a lifeboat if both were equally available? We maintain that just as for general purposes no sane person would tolerate for a moment the substitution of planks for lifeboats whilst lifeboats can be had, so no intelligent and good citizen ought to tolerate, much less to encourage, untrained men in the practice of medicine so long as trained men are available. We insist that in an intelligent state like our own unqualified practice is a crude anachronism, as well as a fraud and a danger, and ought in the public interest to be absolutely stamped out.

The case of Mr. Harness shows quackery at its very best. Harness is a keen and capable man of business, who knows how to put the fairest face upon everything, and who has an incomparable capacity for making the worse appear the better reason. He has proved himself one of those rare persons who, by sheer special endowment, seem to be able "to deceive even the very elect." Yet of this person and his business, defended as he has been by the most strenuous efforts of the bar, a capable magistrate feels himself compelled to affirm "that there was a great deal in the conduct of the business of which no honest man could approve." He said "he could well believe that there were points in the conduct of the business which the management were most unwilling to have exposed to the full light of day." Now, if a business is such that its managers are afraid to expose it "to the full light of day"; if, moreover, it is such that "no honest man" can have anything to do with it, can it be for the interests of the public that such a business should be allowed to be carried on? Let it be remembered that these are the conclusions of a man who is not in a position to form a really competent opinion of the medical aspects and dangers of the business. They are the opinions of an educated man of fair mind on the broad and non-technical aspects of the case. In plain words, quackery, in its most favourable form, has been declared by this fair-minded magistrate to be a thing which will not bear exposure to the "full light of day," and a thing of which "no honest man can approve." If scientists and medical specialists, who alone can see the thing in its true and dangerous light, had been sitting in judgment upon the case, terms of condemnation very different from these would have been truthfully and properly employed.

But if quackery in this favourable form be condemned by all right-minded men, what is to be said of it when practised on the poor and ignorant by unscrupulous persons who have not merely no knowledge of medicine at all, but who are destitute of that general mental capacity and common-sense which enable a man to minimise dangers and to practise the scoundrel's trade with a minimum of scoundrelism? There is only one thing to be said by men who recognise the logic of facts. Quackery ought to be stamped out. A quack medical practitioner is much more dangerous than a mad dog. The law encourages the suppression of mad dogs; it does not, as it now stands, encourage the suppression, but the increase of quacks.

Now let us come to practice. If unlicensed medicine is to be stamped out, who is to lead the way in its suppression? The medical profession undoubtedly. It is mere folly to suppose that lawyers will initiate reforms in the laws which regulate medicine. We ourselves must do that. Loyalty to the State imposes upon us the obligation of uprooting and destroying everything which makes against the health of the State. If it be asked what class of medical men are to undertake this duty? the answer is that the General Medical Council is under the most absolute obligations to lead the way. That Council takes no steps, however, and therefore the whole body of practitioners must organise voluntary societies to effect such alterations in the law as the necessities of the case demand. Of course a certain amount of unpopularity will fall to the lot of any medical man or medical organisation which takes up this line of action, and base motives will be imputed. But is a man or a profession to be deterred from plain duty by the fear of the imputation of unworthy motives? When political Liberals demand reform, somebody is always ready to assign base motives. When Unionists stand for the Union, somebody else sees the cloven hoof of base motives. But does any man, who is a man, hold his hand for that? The medical profession is very timid, and very ready to run away from shadows. That is a kind of cowardice entirely unworthy of educated men, and especially of Britons. The plain duty of doctors is to check and uproot all kinds of ignorant and unscrupulous medicine which endangers the public wellbeing, and that duty they must rise and do, or confess themselves unfit to carry the status and name of Englishmen.

